# Variations in risk perceptions: a qualitative study of why unnecessary urinary catheter use continues to be problematic

**DOI:** 10.1186/1472-6963-13-151

**Published:** 2013-04-26

**Authors:** Molly Harrod, Christine P Kowalski, Sanjay Saint, Jane Forman, Sarah L Krein

**Affiliations:** 1 VA Ann Arbor Healthcare System – HSR&D/CCMR, Ann Arbor, MI, USA; 2 Internal Medicine/General Medicine, North Campus Research Complex, Ann Arbor, MI, USA

## Abstract

**Background:**

Catheter associated urinary tract infection (CAUTI) is one of the most commonly acquired health care associated infections within the United States. We examined the implementation of an initiative to prevent CAUTI, to better understand how health care providers’ perceptions of risk influenced their use of prevention practices and the potential impact these risk perceptions have on patient care decisions. Understanding such perceptions are critical for developing more effective approaches to ensure the successful uptake of key patient safety practices and thus safer care for hospitalized patients.

**Methods:**

We conducted semi-structured phone and in-person interviews with staff from 12 hospitals. A total of 42 interviews were analyzed using open coding and a constant comparative approach. This analysis identified “risk” as a central theme and a “risk explanatory framework” was identified for its sensitizing constructs to organize and explain our findings.

**Results:**

We found that multiple perceptions of risk, some non-evidence based, were used by healthcare providers to determine if use of the indwelling urethral catheter was necessary. These risks included normative work where staff deal with competing priorities and must decide which ones to attend too; loosely coupled errors where negative outcomes and the use of urinary catheters were not clearly linked; process weaknesses where risk seemed to be related to both the existing organizational processes and the new initiative being implemented and; workarounds that consisted of health care workers developing workarounds in order to bypass some of the organizational processes created to dissuade catheter use.

**Conclusions:**

Hospitals that are implementing patient safety initiatives aimed at reducing indwelling urethral catheters should be aware that the risk to the patient is not the only risk of perceived importance; implementation plans should be formulated accordingly.

## Background

Catheter associated urinary tract infections (CAUTI’s) are one of the most commonly acquired health care infections within the United States (U.S.) [1]. Estimates by the Centers for Disease Control stated that, in 2002, 424,060 hospitalized non-intensive care unit patients acquired urinary tract infections resulting in 13,088 deaths [2]. Studies have also found that one in five patients admitted to the acute care setting will receive a urethral catheter during their hospitalization [3]. However, despite published guidelines and evidence-based recommendations, many of the practices to reduce CAUTI are not commonly used [4]. Because of the frequency and associated morbidity with urinary catheter use, there has been a recent focus on initiatives that aim to decrease urinary catheter prevalence and use.

To guide health care providers’ assessments, hospitals are implementing patient safety initiatives aimed at reducing or eliminating risks. Many of these initiatives have been developed at national and state levels and are designed to standardize patient care across hospital settings through disseminating guidelines or formalized efforts to encourage the use of certain practices [5-7]. Such efforts are meant to guide health care providers’ decisions and behaviors thus avoiding placing patients at unnecessary and unacceptable risks. However, recent research has found that “risk” is often locally and, quite possibly, individually defined by health care providers’ and their responses to patient risk are influenced by such things as weak associations between risks and negative outcomes, and organizational processes that support or hinder reducing a patient’s risk for a negative outcome [8,9].

In addition, studies examining the implementation of patient safety initiatives have found that numerous factors influence health care providers’ responses to new initiatives. These factors extend beyond buy-in to evidence-based arguments and include such things as competing priorities or goals, perceived increased workload, activities outside one’s scope of practice, and the determination that evidence-based guidelines are not universally applicable to the targeted patient population [10-12]. All of these factors might be affected by health care providers existing knowledge and experience. As Presseau et al. [10] identified, existing provider behavior might, “influence the performance of guideline-recommended behavior being implemented.”

### Keystone bladder bundle background

A program developed by the Michigan Health and Hospital Association Keystone Center for Patient Safety, known as the Keystone Bladder Bundle (Bladder Bundle) is one initiative aimed at reducing unnecessary urinary catheter use. The specific objectives and components of the Bladder Bundle are described in detail elsewhere [13]. Briefly, the program focuses on the timely removal of urinary catheters and insertion only when indicated [14]. This patient safety initiative is based on the idea that limiting urinary catheter use, primarily by removing the device when it is no longer medically indicated, will decrease CAUTIs. Following a model used previously to successfully reduce central line-associated bloodstream infections (CLABSI) state-wide [15], the Keystone Center acts as a support and resource center providing educational materials and data collection forms, but relies on local hospitals to implement the Bladder Bundle.

The Bladder Bundle was chosen for study because it is a state-wide initiative in which many hospitals, of various organizational structures, size, geographic locations, and (non) academic affiliations, participated. Looking across hospitals, with diverse levels of implementation success, allowed for comparisons to be drawn on how risk is defined, perceived, and acted on in different contexts. Therefore, we sought to answer the following research questions:

•How do health care providers perceive and assess risk?

•How do these perceptions and assessments impact health care providers’ decisions in relation to a patient safety initiative (Bladder Bundle)?

•How does risk become associated with a specific medical device?

## Methods

### Study design, sampling, and data collection

**Figure 1:**
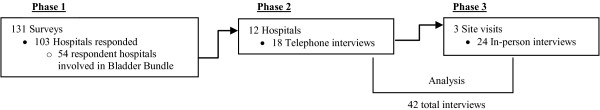
Study design and data collection.

This article focuses on the qualitative components of a larger, sequential mixed methods [16] study whose aim was to understand the implementation of the Bladder Bundle, including such factors as barriers and facilitators, across various hospitals (Figure 1).

In phase 1, a survey was administered to 131 hospitals in Michigan to gather descriptive information on the implementation of practices to prevent health care associated infections (HAIs). Of the 103 hospitals that responded, 54 were involved in the Bladder Bundle and therefore, eligible for participation in the qualitative phases of this study. We used the survey data from these 54 hospitals to construct a maximum variation [17] sample of 12 hospitals based on: (1) number of hospital beds, (2) Bladder Bundle involvement, (3) ease of implementation, as assessed by the local hospital, (4) range of practices implemented, and (5) unit implementing the program.

In phase 2, we conducted 18 telephone interviews with staff from the 12 hospitals. Through snowball sampling [17], we asked each hospital’s infection preventionist to identify 1–2 individuals who had some role in or knowledge of Bladder Bundle implementation. Our semi-structured interview guide focused on understanding how the Bladder Bundle initiative was implemented, barriers and facilitators to implementation, and how effective these hospitals thought they were in changing urinary catheter-related practices.

In phase 3, we again used maximum variation [17] to choose 3 of the 12 hospitals for site visits. Using the survey and corresponding telephone interview data for each site, we chose 3 hospitals that varied in (1) geographic location, (2) bed size, (3) strategies used to implement the Bladder Bundle, (4) barriers to implementation as identified by staff, and (5) other hospital characteristics (for examples, see Table 1).

**Table 1:** Comparison of 3 hospitals chosen for site visits

**Study number**	**Bed size**	**Hospital location and size**	**Practices implemented**	**1) Monitoring catheters placed**	**Units of Bladder Bundle implementation**	**Champion for Bladder Bundle**	**Comments from research team**
				**2) Surveillance for UTI rates**			
Hospital 1	0-150	Rural, community hospital	- Scanner	1) No	Hospital wide	Quality Improvement	This hospital has developed extensive education for nurses and physicians. They have also intervened in the ED. On the floors they use catheter patrols.
			- Aseptic tech	2) Yes, facility			
Hospital 2	151-300	Mid-size referral hospital for surrounding rural area	- Scanner	1) No	Floor only	Nurse and Physician	This site has used both a nurse and a physician as champions. Their reminder states that physicians must order catheter to be continued or nurses will discontinue it.
			- Aseptic tech	2) No			
			- Reminder				
Hospital 3	301-450	Large hospital, suburban, located just outside urban city	- Aseptic tech	1) Yes, facility	Floor only	ICP	They are currently developing their electronic medical record. They do not have a physician champion. Leadership was involved in getting staff on board. Nurses are able to initiate Foley removal.
			- Reminder	2) No			
			- Nurse initiated				
			- Condom catheters				
			- Intermittent catheterization				

We site visited the 3 hospitals and conducted semi-structured interviews with a total of 24 staff, 4 of whom had participated in the telephone interviews. Again, the infection preventionist at each site helped identify interviewees. Interviews were designed to elicit further detail on implementation strategies and processes, and why these hospitals were able to implement changes related to their urinary catheter use.

In sum, because we re-interviewed 4 staff members during our site visits who had also participated in the telephone interviews, our total number of interviews were 42 but only 38 individuals were actually interviewed. All telephone and site visit interviews were audio-recorded and transcribed verbatim by a professional transcriptionist. All of the authors participated in several interviews either conducting or observing them. Institutional review board approval was obtained from the University of Michigan Medical Institutional Review Board and the VA Ann Arbor Healthcare System Human Subjects Committee.

### Data analysis

We aggregated and analyzed the telephone and site visit interviews (N = 42) using open coding and a constant comparative method [17], which entailed coding the transcripts, developing a codebook, and, as the codes evolved and became more refined, returning to the earlier transcripts to ensure that all codes were applied consistently across transcripts. Although data saturation was complete mid-way through the transcripts, we continued to code all interviews to ensure no new codes were present and to document any contrary data. We then analyzed our codes with the supporting data and found that the concept of “risk” played a prominent role in how health care providers were assessing and using the urinary catheter. We then examined the literature to identify, if one existed, a framework that might provide constructs that would explain the associations between risk and health care providers’ decisions to use (or not) the urinary catheter. We identified Dixon-Woods et al. [9] “staffs’ orientations to risk” framework as a means to organize and explain our findings.

### Theoretical framework: staff orientations to risk

In the risk framework described by Dixon-Woods and colleagues [9], they suggest that “staff are routinely engaged in the classification and response to risk. They engage in practices of determining what gets to count as risk, how such risks should be properly managed, and how to account for what they have done.”

The framework identifies four ways that staff orient to risk:

•Normative work- staff deal with competing priorities about matters that are inherently contestable;

•Tightly coupled errors- negative outcome and the error are clearly linked;

•Process weaknesses- risks arise because of fallible and precarious organizational processes;

•Cutting corners- staff acknowledge that they do not always do things perfectly but produce a range of justifications for their behavior.

Dixon-Woods et al. [9] contend that these orientations to risks emerge out of health care providers’ experiences at the “sharp end,” or where the work is actually done and where problems are most likely to arise.

## Results

We applied our codes to the four ways staff orient to risk framework and found that this framework provided an in-depth means of understanding how risk and use of a device are related. We also used contrary data to expand and build upon this framework to include other variables we found that might influence perceptions of and responses to risk. These additions resulted in some expansions and redefinitions of the risk orientations which are explained below.

### Normative work

Dixon-Woods et al. [9] state that staff deal with competing priorities about matters that are inherently contestable. Our data suggests that what might be contestable are other patient safety initiatives. When health care providers related risk to the initiative being implemented, they would prioritize their work because of other, numerous, patient safety initiatives that were often implemented concurrently. On one unit, we heard that they were participating in four separate patient safety initiatives (pressure ulcer, falls, delirium, and medication error prevention). As one participant stated:

… it’s complicated to try to keep that patient safe (Hospital 5- Medical/Surgical Charge Nurse).

In many of the hospitals interviewed, falls were classified as “never-events” and, therefore, were often the primary focus for nurses. In relation to falls, urinary catheters were seen as both a preventive strategy against falls and a potential cause of falls.

Example of urinary catheters preventing falls:

It’s that nurses are worried, “Well do I really want this person hopping out of bed and can I really be sure that they’re going to call me to help them?” We don’t want there to be any falls. That’s considered a never-event in a hospital and we don’t want them to have a [urinary] catheter and, we’re not sure because they just had anesthesia, are they really going to remember to put on their call light or will they try to get up and go to the bathroom? (Hospital 5- Infection Preventionist)

Example of urinary catheters causing falls:

… a lot of times it [catheter] agitates them [patients] more and then they’re trying to climb out of bed because they don’t know the catheter’s there. They keep forgetting and then they feel like they still have to pee, so we had said, you know, ‘Can we pull this catheter, otherwise the catheter’s either going to get pulled out by the patient or they’re trying to get out of bed and they’re going to injure themselves,’ (Hospital 7- Staff Nurse).

The quotes above illustrate how the use of the urinary catheter was perceived differently between two hospitals. In the first case, use of the urinary catheter was seen as a strategy to prevent falls. Thus, urinary catheter use was viewed as compatible with other patient safety initiatives. In the second case, the urinary catheter was seen as a potential cause of falls and, therefore, use of the urinary catheter posed a risk, not necessarily to infection, but to the “never-event” falls.

In addition to prioritizing patient safety initiatives, health care providers were also prioritizing their day-to-day patient care workload. Because nurses were caring for multiple patients, they had to choose which patient care activities were most important and then decide how these activities were going to get done. Many of our interviewees stated that nurses view the urinary catheter as a means to lighten their workload. So, in their opinion, nurses would either ask physicians to order a urinary catheter or they would not ask for an early discontinuation order.

It is hard for a nurse when she’s got five patients, two of which are critical, a patient wants to get up and use the toilet or wants to get on a bedpan and she’s doing six other things, it’s hard for her to break the habit of just going to put in a [urinary catheter] so she can get to the rest of her work. That is a legitimate impediment… (Hospital 11- Infection Preventionist)

However, it should be noted that our findings about nurses not removing catheters or inserting them as a convenience is largely based on the perceptions of those we interviewed (most of whom were management and senior level practitioners) and not based on staff nurses themselves speaking to this issue. What we did find was that when the whole work context was taken into account, it became clear that use of the urinary catheter was possibly in response to other organizational issues, such as insufficient staffing, that increased nurse workload.

In addition to hearing about use of the catheter for “nurse convenience” we also heard that some physicians, mainly those in the Emergency Department (ED), will also insert a catheter for “convenience.”

Dr. XXX went to the physician services meetings, talked to the docs about how important it is not to just do it for convenience because in the [ED] it sometimes is for convenience and in our [ED], typically we could have 25 people waiting for a bed, full with 25. It’s crazy (Hospital 12- Infection Preventionist).

As with the nurse convenience finding, the quote above demonstrates that other issues, such as being too busy to be able to assist a patient to the bathroom, may be contributing to health care providers’ decisions to insert a catheter and only peripherally-related to convenience. The perception that catheters are being inserted for “convenience” may hinder the implementation success of patient safety initiatives because it does not speak to the underlying organizational issues, such as lack of staffing or lack of medical alternatives, (e.g., a bladder scanner that could help determine the need for a catheter) that may be contributing to these decisions.

Many patient safety initiatives, including the Bladder Bundle, were seen by health care providers as competing with one another rather than complementary. Like Dixon-Woods et al. [9], our findings also suggest that health care providers engage in certain work activities based on how they prioritize patient safety initiatives in relation to patient and work outcomes. The “worse” the outcome, (not necessarily to the patient) the higher the priority. Additionally, we found that some patient safety initiatives were seen as “threatening” to other initiatives so health care providers often had to decide which initiative they would focus on.

### Tightly coupled errors (or loosely coupled errors?)

Tightly coupled errors are when the link is made between the error and negative outcomes. The link happens when there is a, “significant lapse in patient safety that [can] be directly attributed to someone doing something incorrectly” [9]. In contrast, we found a lack of tightly coupled errors; what we re-defined as *loosely* coupled errors. Although most participants acknowledged that urinary catheters could cause CAUTI, the issue for some was that this link was not very compelling because the outcome was often not immediate or life-threatening. For example, one interviewee stated:

… so it’s just making them [nurses] understand that there is a relationship between bladder infections and urinary tract infections and [urinary] catheter days… (Hospital 8- Director of Nursing)

One hospital even made it a point to collect urinary tract infection (UTI) data on patients seven days post-discharge to use as evidence for their staff that urinary catheters (and hence their actions related to the use of urinary catheters) do cause infections.

We even track UTI associated with a [urinary] catheter post hospital… We’re trying to get some [infections] to show people, “See? There it is, a UTI occurring. It just didn’t happen quick enough for you to see it in the hospital.” (Hospital 5- Infection Preventionist).

We found that, although increased risk for infection related to urinary catheter use resonated with some health care providers’, other factors also influenced how they viewed this device. For example, some of our participants stated that the urinary catheter is a “low tech” or basic nursing procedure and, consequently, does not pose much risk to the patient. In addition, some nurses and physicians did not see CAUTI as a significant risk to their patients’ health compared to other infections, such ventilator-associated pneumonia (VAP) and CLABSI, which were seen as more risky and, therefore, prioritized above CAUTI.

… it’s difficult to find people that are excited about getting Foleys out of patients; other things take higher priority like central lines and VAP (Hospital 11- Director of Nursing).

The link between urinary catheter use and whether CAUTI represents a significant lapse in patient safety also appears to be influenced by personal experience. For example, we heard that many nurses themselves had experienced UTI’s and, therefore, considered it an easily curable condition. It was basically seen as an “innocuous” infection.

Let’s think about it, the majority of our RNs are still female and they’ve all had hundreds of urinary tract infections in their life time. They did not die (Hospital 2- Infection Preventionist).

Unlike tightly coupled errors, we did not find a link between a lapse in patient safety and someone doing something wrong. In fact, if someone did do something wrong, such as improper insertion that resulted in a CAUTI, health care providers felt that it was easily treatable and, therefore, did not pose much risk. In addition, we found that perceived risk associated with the urinary catheter and the specific outcome of CAUTI was relatively low; what we re-defined as *loosely* coupled errors. The urinary catheter was considered a basic or “routine” procedure and for some health care providers, a part of standard care. The Bladder Bundle tries to clearly link use of the urinary catheter with increased risk for infection to the patient. However, this link was sometimes minimized based on whether providers viewed the severity of CAUTI as a significant patient safety problem and if the risk of catheter use was associated with developing a negative outcome.

### Process weaknesses

According to Dixon-Woods et al. [9] process weaknesses are organizational processes that health care providers’ believe could pose more of a risk when used. They found that when a process did fail, it was unclear who had the authority to change the process and, therefore, the suboptimal process usually continued causing health care providers’ to work in “reactionary” mode.

However, our data led us to reconceptualize the notion of process weaknesses to include the initiative being implemented. Where Dixon-Woods et al. [9] concept focuses on how organizational processes can pose a risk if used, we found that problems can also arise if the initiative is not context appropriate. We found that, when implementing this initiative, hospitals experienced difficulties due to the disparity between the Bladder Bundle’s processes for use and the pre-existing organizational processes. Hospitals in our study often experienced two process weaknesses that were difficult to overcome; (1) the context in which they were trying to implement the Bladder Bundle (organizational process weakness) and; (2) the indications for urinary catheter use (Bladder Bundle process weakness).

The first process weakness was how hospitals were trying to apply the Bladder Bundle indications in organizational settings and populations for which the program was not designed. For example, more specialized units, such as obstetrics (OB), had difficulty with the Bladder Bundle indications because what they used the urinary catheter for was either not listed or did not seem to apply to their patients. In one hospital, it was organizational policy that if an OB patient had an epidural they automatically received a urinary catheter which is not a Bladder Bundle indication. Therefore, to not insert a catheter would go against hospital policy.

… one of our challenges is what to do with epidurals and our culture is that if you have an epidural in place, you have a [urinary catheter] in place and [health system] as a whole has decided not to tackle that too much yet (Hospital 6- Clinical Nurse Specialist).

What was also interesting was that there was recognition, by the hospitals, that the indications did not seem to be developed for specialized units, but they went ahead with trying to implement the initiative anyway.

. . . I looked at the criteria set forward indications for [urinary] catheter use. I think my gut reaction was that perhaps some of those were not as applicable in the ED setting, that maybe they were more devised for the inpatient setting (Hospital 12- Infection Preventionist).

The second process weakness, indications for urinary catheter use, as defined by the Bladder Bundle initiative, created difficulty when hospitals tried to rigidly apply these indications. The Bladder Bundle has a list of indications, based on expert opinion, that are meant to guide health care providers in deciding whether or not a urinary catheter is necessary and appropriate. However, we found that health care providers continued to use the urinary catheter for non-indicated reasons.

One of the most cited reasons for use of the urinary catheter was to determine Intake’s and Output’s (I’s and O’s) and this measurement was seen, by some health care providers, as “common” practice. A patient’s fluid balance is monitored carefully through I’s and O’s. This measurement is considered a management tool that provides information on a patient’s hydration level, and renal and cardiovascular function. However, within the Bladder Bundle program, I’s and O’s are not an indication for urinary catheter use except for critically ill patients. Also, there is a lot of debate both as to the usefulness of this measurement for monitoring a patient’s condition and for acceptable alternatives. For example, one participant stated that she could not get consensus among the physicians on an alternative measurement. Weight was suggested by the Bladder Bundle but many physicians felt that this measurement was not accurate:

I think it’s [I’s and O’s] still an issue . . . it’s hard to define. The (Bladder Bundle) Project, said you’re supposed to be using the weight really more than the measure, etcetera. But that is a hard thing to actually get everybody to agree upon and to practice (Hospital 12- Infection Preventionist).

Physicians felt that the alternative measurement for I’s and O’s, weight, was not accurate enough and, therefore, continued to use the catheter. We cannot conclude, by virtue of our findings, that physicians viewed not using the catheter as more of a risk. The relationship between risk and *not* using a device is an area that needs further exploration.

One hospital thought the Bladder Bundle indications were so ambiguous and inapplicable to their patient population, they developed their own indications:

To be honest with you, the reasons [indications] why the catheters were in, that was very confusing, that needs to be tightened up… I asked for a little more clarification, didn’t really get it, so we kind of developed our own… (Hospital 3- Project Manager, Quality and Research)

In addition, no matter how explicitly the Bladder Bundle indications were stated, interpretations of these indications occasionally differed from what was intended. For example,

This is kind of a weird thing that happened but one of the appropriate indications is prolonged immobilization and I think the intent of it was a patient who had a thoracic or lumbar spine fracture that was unstable. However, I think many people are selecting that indication just because patient’s going to be on bed rest (Hospital 6- Trauma nurse specialist).

Although more work in this area is warranted, by reconceptualizing Dixon-Woods et al. construct [9], we found that process weaknesses may have to do with both the pre-existing organizational processes and the initiative being implemented. Some units were able to directly relate urinary catheter use to the Bladder Bundle indications whereas other units found it difficult to apply these indications to both their patient populations and the units existing context. Ultimately, staff were trying to implement the Bladder Bundle’s processes into contexts that were a poor fit due to pre-existing organizational processes. In fact, in one hospital, non-use of the catheter went against their policy. In addition, the Bladder Bundle’s indications were based on “expert opinion” but these indications did not address the existing behaviors as to why the catheter was being used in the first place. We also found that when the Bladder Bundle tried to restrict the use of the urinary catheter through “appropriate indications,” health care providers developed workarounds to continue to use the urinary catheter in ways they deemed appropriate.

### Cutting corners (or workarounds?)

As described in the Dixon-Woods et al. framework [9], when staff were involved in managing risks, they would often “cut corners,” meaning not follow the standardized procedures, and then justify the reasons for such noncompliance. Justifications included not relating the behavior or activity to infection, questioning the standards, and viewing such behavior as the “norm” because they were following what co-workers were doing.

However, our findings suggested that health care providers were more likely to engage in *workarounds* then cut corners. Workarounds and related patient safety incidents have been studied in various health care settings [18-21]. The concept of workarounds is generally thought of as, “work procedures that are undertaken to bypass perceived or real barriers in work flow,” [19]. We found workarounds involved several of the strategies implemented to limit use of the urinary catheter, including those related to the use of technology, assessment for catheter need, and ongoing monitoring of catheter use.

We found that the electronic medical record (EMR) offered health care providers several opportunities to by-pass organizational processes (based on Bladder Bundle recommendations), to continue to use the urinary catheter. Documentation, technological or written, often did not reflect why urinary catheters were being used. For example, use of an “other” category in the EMR ordering system as the indicated reason for urinary catheter use was common at one site but this made it difficult to both understand the reasons for insertion and to change the resulting behavior.

I think some of the challenges are related to our electronic medical record, the way it’s set up because when a physician goes in to order a catheter, they put in the order and then they have to select one of those, I think there are 7 indications and it’s all the approved indications. But the way our rules are with our electronic record, we always have to give physicians something of an out, that if it doesn’t fit in those categories, they can select “other” or they can bypass it (Hospital 6- Trauma Nurse Specialist).

Some hospitals were implementing electronic orders to standardize the various uses of the urinary catheter indications and assessments. However, this solution presented its own set of workarounds. For example, one hospital’s EMR allowed physicians to set up their “favorites” in their order sets where indications were preselected. Therefore, the urinary catheter was deemed “appropriate” even if it might not reflect the actual reason for use.

Another workaround we found had to do with urinary catheter assessments. Once the urinary catheter is inserted, the Bladder Bundle requires “necessity” assessments to be done to determine the on-going need for the urinary catheter. The purpose of these assessments is to ensure that unnecessary urinary catheters are removed promptly thus decreasing the likelihood of CAUTI. We found, however, that these assessments were used as workarounds because health care providers had developed variations in how they interpreted and applied these assessments. For example, at one site that had developed an automated system and scoring algorithm to indicate whether the urinary catheter was still necessary, one participant stated:

I wouldn’t say it’s [needs assessment] been as helpful as we had hoped it would be because I find that sometimes they forget to do it and sometimes they’ll just mark something that gets the patient a 5, [5 indicates that a urinary catheter is needed] unfortunately . . . we still have some nurses who don’t want to deal with it so they’ll just put “reevaluate in one week” or whatever (Hospital 2- Clinical Nurse Specialist).

Like the documentation issues, this scaled necessity assessment, which was intended to aid health care providers’ in their decision making process, was being used to justify keeping a urinary catheter in, thus it did not change the underlying behavior.

An additional workaround was the use of “catheter patrols.” Catheter patrols consisted of health care providers checking each inpatient for a urinary catheter, if appropriate indications were documented, and whether or not the urinary catheter was still necessary. Use of the catheter patrols was meant to prompt the physician or nurse for a removal order if the urinary catheter was found to be unnecessary. Catheter patrols were often headed by someone other than the bedside nurse such as the infection preventionist or the unit manager. Catheter patrols were successful in decreasing extended urinary catheter use but, when these catheter patrols ceased, urinary catheter use often increased indicating that health care providers were responding to the catheter patrol and not the patient safety argument.

. . . now you can tell why that catheter patrol was so essential though because they took the catheter patrol away briefly. And our rates went up… I think they took it away for 2–3 months thinking that things were going to be on autopilot and they were not. I think we’ll need a permanent catheter patrol (Hospital 3- Family Practice Physician).

The catheter patrol was used as a workaround on the nursing side of care because, instead of the bedside nurse taking responsibility for the daily assessment and follow-up with physicians, this activity was taken on by someone else who, perhaps, was not directly involved in patient care. Therefore, the bedside nurses were not prompted to change their behaviors and integrate this initiative into their everyday work.

In comparison to Dixon-Woods et al. [9] cutting corners, we found that health care providers were more likely to develop *workarounds* to complete their work tasks. Even with the use of the EMR, health care providers were still able to find ways to work around its structure and continue to use potentially unnecessary urinary catheters. The Bladder Bundle “necessity” assessment was pliable enough it would actually support behavior it was meant to change. The catheter patrols, even though designed to get health care providers to think differently about catheters, only managed to control their behavior when it was patrolling.

## Discussion

Our findings suggest that health care providers use diverse understandings of risk when they are assessing the use of the urinary catheter. Many of our findings correlate with Dixon-Woods et al. [9] “four ways staff orient to risk” framework. However, some of our findings were contrary to the risk framework’s constructs and, therefore, we were also able to expand and build on these orientations to better understand how risk comes to be (or not) associated with certain medical devices and how that influences health care providers’ decisions. Based on our findings, we re-defined two (tightly coupled errors and cutting corners) of Dixon-Woods et al. [9] risk orientations which provides additional conceptions of risk that health care providers’ may be applying when making healthcare decisions regarding urinary catheter use.

Similar to Dixon-Woods et al. [9] normative work construct, we found that health care providers often prioritized their work according to which patient safety initiatives were being emphasized at a hospital, unit, and individual level. Expanding on normative work, our findings suggest that how work gets prioritized is dependent on various factors including perceived compatibilities between patient safety initiatives and perceived risks of doing these initiatives. Understanding these factors requires asking what, why, and how initiatives are viewed and prioritized among health care providers’ on the units. This type of approach opens up a number of explanations which go beyond evidence-based arguments and might resonate more with health care providers who are tasked with doing the initiatives.

Even though the Bladder Bundle’s approach is focused on relating the use of the urinary catheter to increased risk for infection, we found that this was not always the main source of information health care providers’ used for medical decision making. We found that clinical and personal experiences also became part of the equation. The integration of these experiences into the decision making process resulted in what we re-defined as loosely coupled errors where the cause and the outcome were not clearly linked or, at least, thought to be relatively innocuous. Education based on the relationship between use and increased risk for infection did not seem to engage health care providers enough to change their behaviors. Addressing their concerns might be a more effective approach. These concerns might be how to prioritize and integrate work, offering alternatives (e.g., condom catheters) that are seen as just as effective or “better” than the urinary catheter, and promoting patient safety initiatives as compatible with one another so health care providers can manage them as one process. For example, one hospital that was focusing on preventing falls instituted hourly bathroom rounding which was compatible with not using urinary catheters. Another hospital that had been focusing on pressure ulcers integrated the Bladder Bundle by demonstrating how the absorbent pads they were using were also an alternative to using a urinary catheter.

We reconceptualized Dixon-Woods et al. [9] construct of weak organizational processes to include new initiatives that are implemented in various health care settings. We found that when local hospitals tried to implement the Bladder Bundle program, using the criteria provided to define appropriate indications for urinary catheter use, many of the “weaknesses” became apparent. Although these indications were thought to be clearly defined, there seemed to be a lot of ambiguity in how they were interpreted and applied by health care providers. Furthermore, even though the initiative was intended for specific units/populations, no guidance was given as to how to adapt it for different contexts. Organizations that develop patient safety initiatives either have to be very explicit about the environment in which the initiative is to be implemented or the initiative might have to be developed with flexibility and adaptability in mind to support broader implementation contexts. Also, existing organizational policy that contradicts the initiatives recommendations should be examined to determine if the policy makes more sense given the context.

We re-characterized cutting corners as workarounds to better represent and explain our findings. Similar to previous research [18-21], we found that workarounds resulted when health care providers could not integrate the Bladder Bundle into their everyday work. The technology that was developed to prevent variations in urinary catheter use often stripped the users of their tacit knowledge and forced them into decisions based on categories. When these categories did not fit the context, health care providers developed workarounds to ensure that they could continue providing patient care in ways they deemed important. In addition, although the “catheter patrol” was meant to monitor the use of the urinary catheter and ensure that unnecessary urinary catheters were removed, the patrol was only successful when it was in operation and sustained success was not achieved. Health care providers were able to find ways to bypass the organizational and Bladder Bundle processes in order to continue their established workflows. These workarounds made it difficult to understand where the implementation difficulties resided; either at the organizational level, the individual level, or in the interaction between the two.

Dixon-Woods et al. [9] framework provided additional insight into how health care providers’ assessed risk and the effect this might have on their subsequent care decisions. We were able to relate and expand on this framework to include additional explanations for why health care providers might or might not participate in patient safety initiatives. The concept of risk is important to understand because, as we found, it does impact implementation.

This research further informs the development and evaluation of new initiatives by identifying the concept of “risk” as a potentially key factor in understanding how initiatives come to be accepted, rejected, or adapted to fit the context. Our findings suggest that it is important for implementation efforts to incorporate approaches that elicit health care providers existing ways of thinking and how new initiatives are asking them to change those lines of thought. Application of this knowledge might then be used to move health care providers toward the wanted practice change.

Our research was limited by its focus on one patient safety initiative of one device. It is difficult to say if these findings relate to other initiatives and/or medical devices. In addition, some of the hospitals had participated in a very successful ICU initiative that was also developed by Keystone. Their involvement might have affected their perceptions of success and judged the Bladder Bundle more negatively then if they had not participated in the ICU initiative. However, because hospitals are continually implementing quality initiatives to various levels of success, we believe that, regardless of their involvement with the ICU initiative, these hospitals would have struggled with implementation because of other factors we found to be significant. Another limitation is that we were not able to interview as many staff nurses as we would have liked mainly due to their work schedules and the burden it places on the unit when they are removed from the floor. Since staff nurses are often tasked with implementing patient safety initiatives, their experiences are needed and would greatly add to this literature.

Future research should involve different patient safety initiatives to determine what other factors affect how risk is perceived and how these perceptions impact patient care and the implementation of patient safety initiatives. In addition, the development and testing of an assessment tool to gather various interpretations of risk would aid in comparisons being drawn across initiatives and contexts. Such an assessment tool could then be used to mitigate health care providers’ perceptions of risks and help move behavior toward the desired outcome.

## Conclusions

The challenge is to develop patient safety initiatives that fit seamlessly into already established work processes. Developing such a program requires a full understanding of how health care providers interpret risk, assess patients for such risks, and how this impacts patient care decisions. This could entail pre-implementation assessments of how health care providers use a specific device and their reasons for such use. Then, a program that addresses these issues could be developed with the understanding that one hospital might have to use various approaches depending on different health care providers’ interpretations of risk. Even though the goal might be patient safety, the actual process of getting there must include a better understanding of how the concept of risk influences health care providers’ behaviors and activities.

## Competing interests

The authors declare that they have no competing interests.

## Authors’ contributions

MH made substantial contributions to the design, acquisition, analysis and interpretation of data, and was the primary writer of the manuscript. CPK made substantial contributions to the design, acquisition and analysis of the data and was involved in the revisions of the manuscript. SS made contributions to the design and acquisition and to revising the manuscript for important intellectual content. JF made important contributions to the design of the study and to the revision of the article for important intellectual content. SLK made substantial contributions to the design, acquisition, analysis and interpretation of data, and was critical in the design of the manuscript development. All authors read and approved the final manuscript.

## Pre-publication history

The pre-publication history for this paper can be accessed here:

http://www.biomedcentral.com/1472-6963/13/151/prepub
